# Using proximity extension proteomics assay to identify biomarkers associated with infarct size and ejection fraction after ST-elevation myocardial infarction

**DOI:** 10.1038/s41598-020-75399-6

**Published:** 2020-10-29

**Authors:** Moman A. Mohammad, Sasha Koul, Anna Egerstedt, J. Gustav Smith, Marko Noc, Irene Lang, Michael Holzer, Peter Clemmensen, Olof Gidlöf, Bernhard Metzler, Thomas Engstrøm, David Erlinge

**Affiliations:** 1 Department of Cardiology, Clinical Sciences, Lund University, Skane University Hospital, Lund, Sweden; 2 Center for Intensive Internal Medicine, Ljubliana, Slovenia; 3 Department of Cardiology, Medical University of Vienna, Vienna, Austria; 4 Department of Emergency Medicine, Medical University of Vienna, Vienna, Austria; 5 Department of Cardiology, University Heart and Vascular Center, Hamburg, Germany; 6 Division of Cardiology, Department of Medicine, Nykoebing F Hospital, University of Southern Denmark, Odense, Denmark; 7 Department of Cardiology, Innsbruck, Austria; 8 The Heart Center, Rigshospitalet, University of Copenhagen, Copenhagen, Denmark

**Keywords:** Predictive markers, Prognostic markers, Cardiovascular biology, Interventional cardiology

## Abstract

Plasma concentrations of many cardiovascular and inflammatory proteins are altered after ST-elevation myocardial infarction (STEMI) and may provide prognostic information. We conducted a large-scale proteomic analysis in patients with STEMI, correlating protein levels to infarct size and left ventricular ejection fraction (LVEF) determined with cardiac magnetic resonance imaging. We analysed 131 cardiovascular and inflammatory proteins using a multiplex proximity extension assay and blood samples obtained at baseline, 6, 24, and 96 h from the randomised clinical trial CHILL-MI. Cardiac magnetic resonance imaging data at 4 ± 2 days and 6 months were available as per trial protocol. Using a linear regression model with bootstrap resampling and false discovery rate adjustment we identified five proteins (ST2, interleukin-6, pentraxin-3, interleukin-10, renin, and myoglobin) with elevated values corresponding to larger infarct size or worse LVEF and four proteins (TNF-related apoptosis-inducing ligand, TNF-related activation induced cytokine, interleukin-16, and cystatin B) with values inversely related to LVEF and infarct size, concluding that among 131 circulating inflammatory and cardiovascular proteins in the acute and sub-acute phase of STEMI, nine showed a relationship with infarct size and LVEF post-STEMI, with IL-6 and ST2 exhibiting the strongest association.

## Introduction

Myocardial infarction is a consequence of atherosclerosis, and chronic inflammation has long been thought to contribute to the progression and instability of atherosclerotic plaques. However, causality was not proven until recently with the Canakinumab Anti-inflammatory Thrombosis Outcome Study (CANTOS)^1^. In the CANTOS trial, canakinumab, an interleukin-*β1* inhibitor, resulted in lower rates of nonfatal MI, stroke, and cardiovascular death without lowering lipid levels. Although methotrexate did not impact clinical outcome in the Cardiovascular inflammation reduction trial (CIRT), the recently-published results of the Colchicine Cardiovascular Outcome Trial (COLCOT) and Colchicine in Patients with Chronic Coronary Disease Trial (LoDoCo2) further support this concept^2–4^. However, the influence of inflammation in myocardial infarction extends beyond chronic inflammation, as acute inflammatory processes are believed to play a role in reperfusion injury and tissue repair in the infarcted myocardium. This is suggested by observational and experimental studies showing elevation of a number of inflammatory and cardiovascular proteins in the acute phase of MI^5^, correlating with infarct size, particularly in the setting of reperfusion^6–8^. Current understanding is that a balance between inflammation and its resolution, the result of fine-tuned interplay between pro-inflammatory and anti-inflammatory proteins, is central to adequate tissue healing and salvage of compromised myocardium^9^. Excessive inflammation is thought to contribute to tissue remodelling, fibrosis, and scarring of the heart^10^, whereas a lack of inflammation may result in inadequate tissue healing^11,12^.

Biomarker studies provide an important route to understanding myocardial injury, which is vital to development of innovative therapies. However, few studies have assessed the relative importance of proteins by assessing a large number of circuating proteins simultaneously^13–15^.

The objective of this study was to perform a large-scale proteomic analysis using a novel proximity extension assay (PEA) to evaluate the hypothesis that plasma concentrations of 157 inflammation- and cardiovascular-associated proteins correlate to infarct size and ejection fraction in the acute and chronic phase post-STEMI as assessed by cardiac magnetic resonance imaging (CMR).

## Materials and methods

### Study population and design

All patients from the Randomized Controlled Study of the Use of Central Venous Catheter Core Cooling Combined With Cold Saline as an Adjunct to Percutaneous Coronary Intervention for the Treatment of Acute Myocardial Infarction (CHILL-MI) trial^16^ were included in this post-hoc analysis. The study design for the CHILL-MI trial has been previously published^16^. In brief, it included patients with STEMI who underwent percutaneous coronary intervention (PCI) from July 2011 through March 2013 at nine sites in four countries. Participants were randomized to hypothermia induced by rapid infusion of cold saline and endovascular cooling or to standard care. Patients with cardiac arrest, previous myocardial infarction (MI), previous PCI or coronary artery bypass grafting, known congestive heart failure, end-stage kidney disease or hepatic failure, recent stroke, coagulopathy, pregnancy, or Killip class II to IV at presentation were excluded. One hundred twenty patients were randomized and 117 underwent PCI. The primary endpoint in CHILL-MI was infarct size/myocardium at risk assessed by CMR on day 4 ± 2, which was not significantly reduced by hypothermia (relative reduction 13%, *p* = 0.15) and all patients were followed-up with a second CMR after 6 months. Blood samples used in this biomarker study collected at baseline, 6, 24, and 96 h post-PCI were available from 119 patients. The protein profile was analysed by Olink Bioscience (Uppsala, Sweden). The co-primary study endpoints were infarct size (% of left ventricular mass) and ejection fraction (%) assessed in the acute phase (within 4 ± 2 days) and non-acute phase (after 6 months). The area under the curve value for each biomarker was established relative to early infarct size (4 ± 2 days post-MI) and long-term ejection fraction (6 months post-MI) as the co-primary analyses. Secondary analyses included the assessment of ∆ ejection fraction, ∆ stroke volume, ∆ left ventricular mass, myocardial salvage index (%) and microvascular obstruction (expressed as % of left ventricular mass).

The Ethics Committee of Lund University, in agreement with the declaration of Helsinki, approved the study. All participants provided informed consent. This study adheres to the REMARK guidelines for biomarker studies.

### Imaging

A total of 101 patients underwent CMR on day 4 ± 2 days and 86 at 6 months post-STEMI. Reasons for dropout of CMR are available^16^. After scout images to locate the heart and the standard imaging planes, 0.2 mmol/kg body weight of an extracellular gadolinium-based contrast agent was administered. For visualization of the MaR and evaluation of LVEF, early contrast-enhanced steady-state free precession cine images were obtained approximately five minutes after contrast injection. For infarct visualisation, late gadolinium enhanced images were acquired 15–20 min after administration of the contrast agent. Cine and late gadolinium enhanced images were acquired in the short-axis view, from base to apex, and in three standard long-axis views (two-chamber, four-chamber, and left ventricular outflow tract views), in a breath-hold image sequence. The analysis of ventricular dimensions, MaR, and infarct size was performed by the core lab (Imacor AB, Lund, Sweden) using post-processing software (Segment, v. 1.9 R3084; https://segment.heiberg.se). Infarct size was available for 97 of 101 patients at 4 ± 2 days and 82 of 86 at 6 months and is expressed as a percentage of the left ventricular myocardium. Ejection fraction was available for all patients that underwent CMR. Observers blinded to all other data conducted the assessment of infarct size and ejection fraction.

### Assay method

Whole blood samples were centrifuged on site within 1 h, and plasma was separated and stored at − 80 °C in aliquots of 100 μL. One hundred μL of plasma EDTA samples was sent to the core laboratory to be analysed, and 1 μL was prepared according to the manufacturer’s instruction and analysed using a high-throughput technique: Proseek Multiplex CVD I96 × 96 and Proseek Multiplex Inflammation I96 × 96. The PEA assay design has been described in detail^17^. Briefly, 1 μL plasma samples were mixed with 3 μL incubation mix containing pairs of highly specific oligonucleotide-labelled antibodies for each target protein. The oligonucleotides were subsequently joined using a DNA polymerase, and the PCR template was extended, after which uracil-DNA glycosylase was added, digesting the DNA templates and the remaining universal primers. Sample mix was quantified by microfluidic real-time PCR, and protein values were converted to normalized protein expression units on a log_2_-scale in which protein values indicate concentration as opposed to absolute quantity.

Laboratory personnel blinded to patient characteristics, clinical outcomes, and treatment allocation performed all biochemical analyses. The limit of detection (LOD) was determined for each protein biomarker based on the mean value of negative controls plus 3 standard deviations calculated from large datasets by Olink Bioscience. Standard curves for all target proteins are available online (https://www.olink.com/products/complete-protein-biomarkers-list/). Biomarkers with missing values or values below the LOD exceeding 25% of all measurements were excluded from the primary analysis: IL17a, IL20Ra, IL2rb, IL1alpha, TSLP, IL2, IL10Ra, IL22Ra1, PDL1, IL24, IL13, ARTN, TNF, IL20, IL33, IFN-gamma, IL4, LIF, NRTN, ST1a1, IL5, ITGB1BP2, MAMP, BNP, and PSGL1. Due to the well-established association of NT-proBNP to ejection fraction, NT proBNP was excuded. For markers included in the final analysis, values below LOD were replaced by LOD/2. A list of all markers, abbreviations, distribution, LOD, lower limit of quantification, upper limit of quantification, and inter- and intra-assay variation are presented in Supplementary Table S1.

### Statistical analysis

Normality of distribution was assessed from visual inspection of histograms. Normally distributed continuous variables are expressed as means with standard deviation and non-normally distributed continuous variables are expressed as medians with interquartile range (IQR). A linear regression model with bootstrap resampling with 1000 replications was used as the primary statistical model to assess the relation between protein to endpoint. Univariable as well as multivariable analyses adjusting for age, sex, and body mass index along with treatment group (hypothermia or standard care) were conducted. Adjustment for false discovery rate in multiple testing was made using the Benjamini–Hochberg method. A two-sided *p* value < 0.05 was considered significant. All statistical analyses were performed using Stata v. 14.1 for Macintosh, StataCorp, Texas) and figures generated in R v.3.2.2 for Macintosh (R Foundation for Statistical Computing, Vienna and.

## Results

### Baseline characteristics

Patient characteristics and CMR results are presented in Table 1. The mean age on admission was 57.5 ± 10.0 years, and 82% were male. The culprit artery was the right coronary artery (RCA) in 47.1% and left anterior descending artery (LAD) in 42% of patients. A total of 91.6% had TIMI flow grade 3 after PCI.

**Table 1:** Clinical, angiographic, and cardiac magnetic resonance (CMR) imaging data for all patients and those who underwent CMR at 4 ± 2 days and 6 months.

	All patients	Cardiac magnetic resonance imaging 4 ± 2 days	Cardiac magnetic resonance imaging 6 months
	n = 119	n = 101	n = 86
Age (years)	57.5 ± 10.0	57.7 ± 10.4	58.4 ± 10.1
Male	98 (82.3%)	85 (84.2%)	75 (87.2%)
Female	21 (17.7)	16 (15.8%)	11 (12.8%)
Body mass index (kg/m ^2^)	27.6 ± 4.1	27.4 ± 3.8	27.3 ± 3.7
Smoker	51 (42.9%)	42 (41.6%)	35 (40.7%)
Former smoker	30 (25.2%)	26 (25.7%)	22 (25.6%)
Diabetes mellitus	11 (9.2%)	9 (8.9%)	8 (9.3%)
Hypertension	34 (28.6%)	28 (27.7%)	26 (30.2%)
Anterior wall infarction	51 (42.9%)	41 (40.6%)	34 (39.5%)
Inferior wall infarction	68 (57.1%)	60 (59.4%)	52 (60.5%)
Initial TIMI flow grade 0–1	101 (84.9%)	85 (84.2%)	74 (86.0%)
TIMI flow grade 3 after PCI	109 (91.6%)	96 (95.1%)	82 (95.4%)
**Culprit artery**			
Left anterior descending artery	50 (42%)	40 (39.6%)	33 (38.4%)
Right coronary artery	56 (47.1%)	49 (48.5%)	42 (48.8%)
Left circumflex artery	12 (10.1%)	11 (10.9%)	10 (11.6%)
Left main artery	1 (0.8%)	1 (1.0%)	1 (1.2%)
**Cardiac magnetic resonance imaging**			
Infarct size (% of LVM)		18 ± 10	11 ± 6
Infarct size (g)		23 ± 15	13 ± 9
Ejection fraction (%)		48 ± 9	51 ± 10
Myocardium at risk (% of LVM)		37 ± 11	37 ± 11

### Primary endpoints

**Figure 1:**
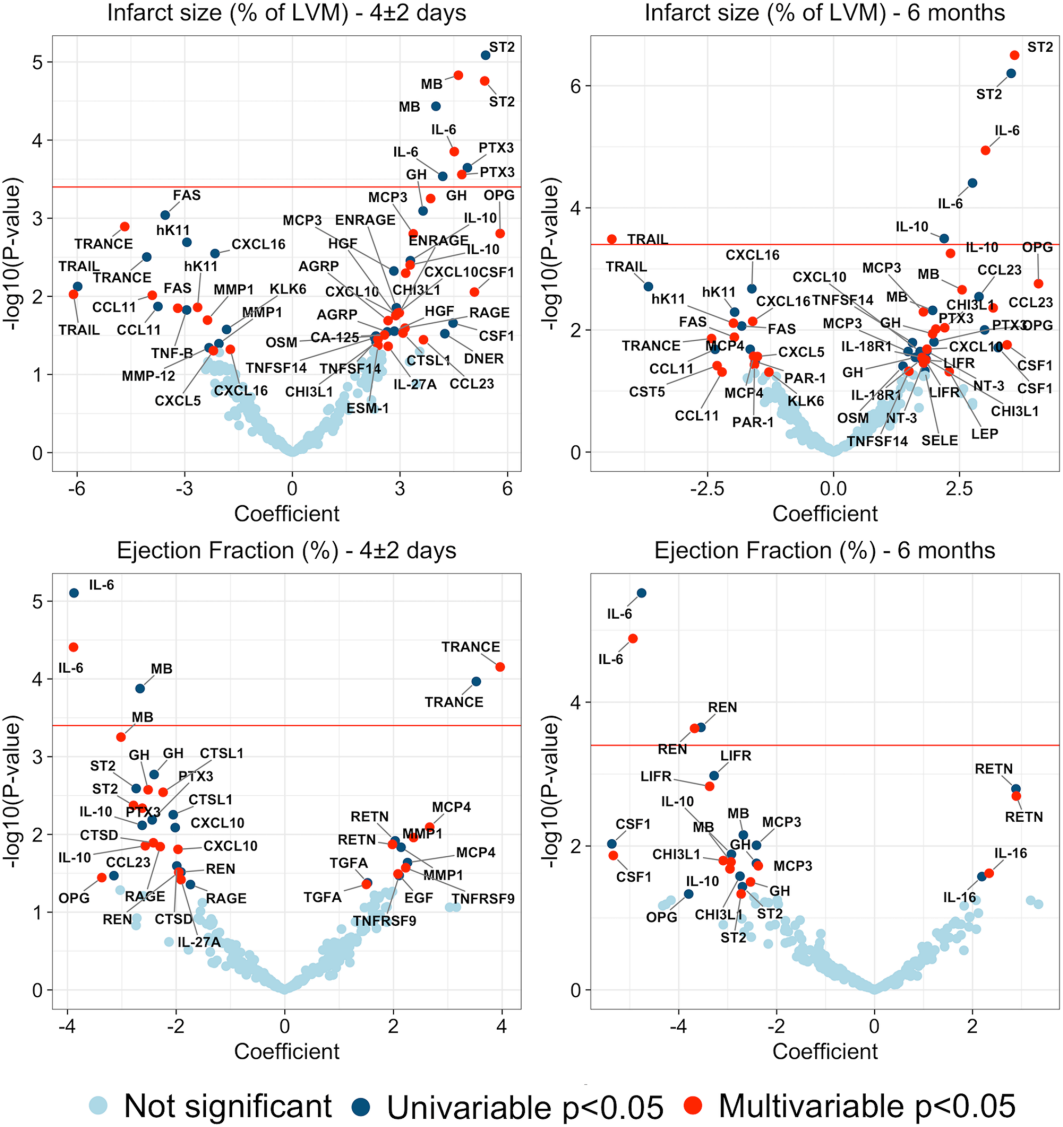
Proteins and primary endpoints. Results of univariable and multivariable regression models for infarct size and ejection fraction at 4 ± 2 days and 6 months. Proteins above the red line remained statistically significant after adjusting for multiple testing.

Several proteins were associated with the study outcomes. ST2, myoglobin (MB), interleukin-6 (IL-6), and pentraxin-3 (PTX3) were significantly positively correlated with infarct size at 4 ± 2 days in both univariable and multivariable analyses adjusted for age, sex, and body mass index as well as treatment arm (Fig. 1). Only ST2 and IL-6 were significantly related to both infarct size at 4 ± 2 days and infarct size at 6 months. A negative correlation of TNF-related apoptosis-inducing ligand (TRAIL) and a positive association of IL-10 with infarct size at 6 months was observed. Univariable and multivariable analyses at 4 ± 2 days and 6 months revealed an inverse relationship of IL-6 with ejection fraction (Fig. 1). TNF-related activation-induced cytokine [TRANCE, also known as receptor activator of the nuclear κ B ligand (RANKL)] showed a positive relationship with ejection fraction in univariable and multivariable analyses at 4 ± 2 days but not at 6 months (Fig. 1). Renin was negatively associated with ejection fraction at 6 months but not at 4 ± 2 days.

### Secondary endpoints

**Figure 2:**
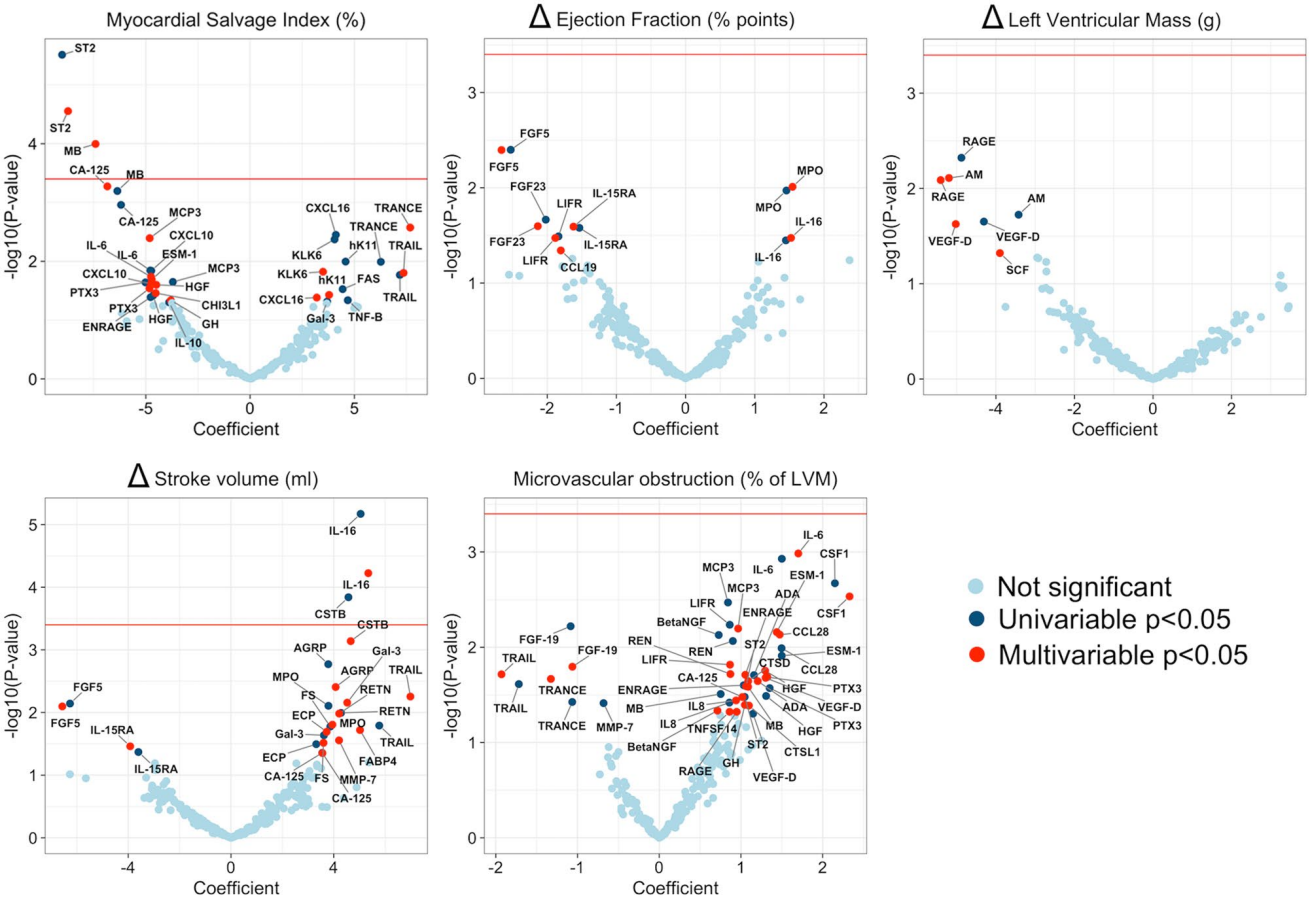
Proteins and secondary endpoints. Results of univariable and multivariable regression models for remaining CMR endpoints. Proteins above the red line remained statistically significant after adjusting for multiple testing.

Two proteins, ST2 and MB were negatively associated with myocardial salvage index (Fig. 2). Borderline significance was observed for cancer antingen-125 where high values correlated with lower myocardial salvage index. After adjusting for multiple testing, no protein was significantly associated with ∆ EF, ∆ left ventricular mass, or microvascular obstruction, but interleukin-16 (IL-16) was positively correlated with stroke volume in univariable and multivariable analyses, and cystatin B (CSTB) was associated with improved stroke volume in the univariable model (Fig. 2).

### Plasma protein concentration profile

**Figure 3:**
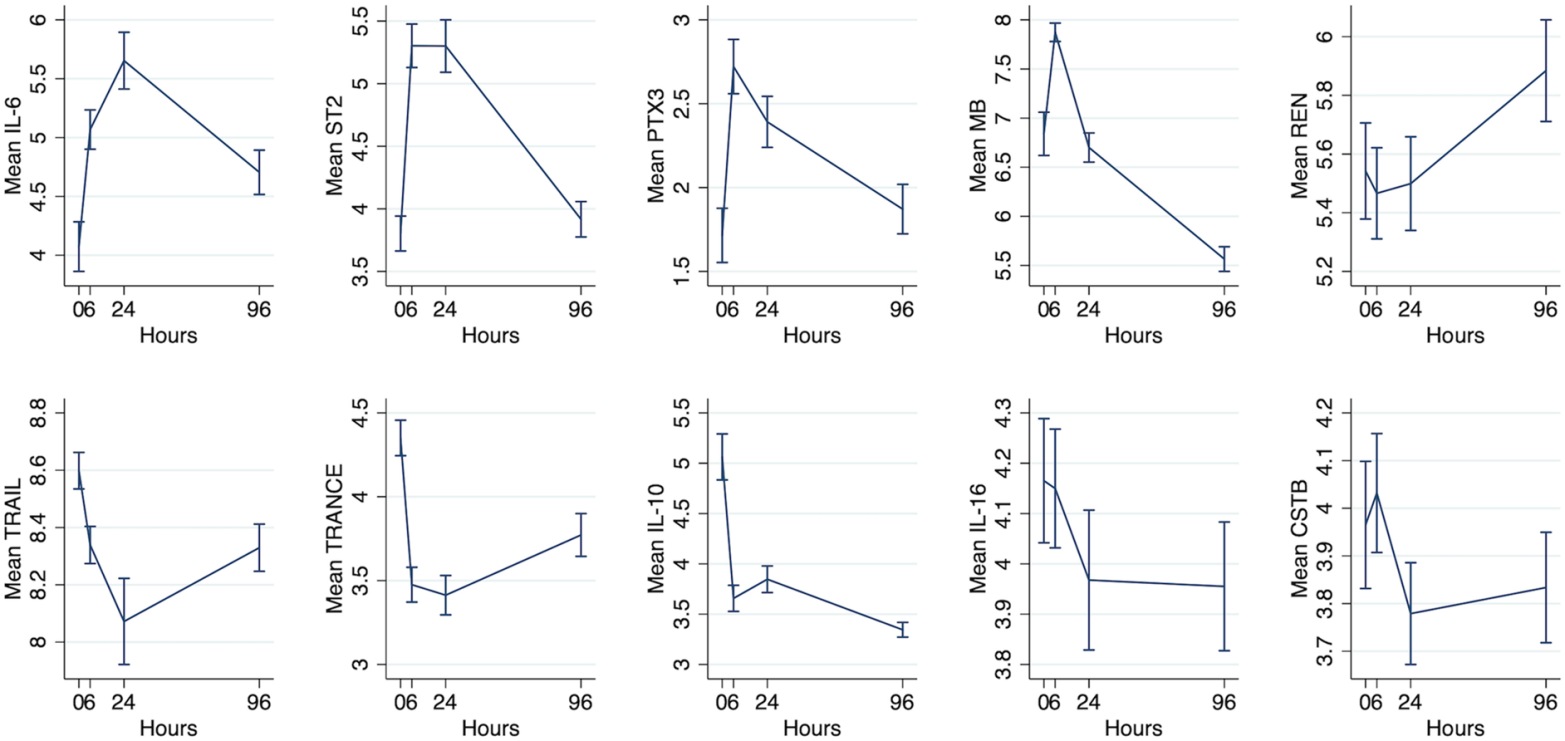
Time–concentration curve showing the release profiles for proteins that were statistically significantly related to endpoints after adjustment for multiple testing.

Time-concentration curves of proteins significantly associated with primary and secondary endpoints are shown in Fig. 3. Proteins that were significantly related to outcome measures after FDR adjustment showed an early release profile followed by either a steady increase or decrease. Proteins associated with larger infarcts and lower ejection fraction increased in concentration shortly after PCI, while, in contrast, proteins associated with a favourable outcome decreased in concentration in the hours after MI, with the exception of IL-10.

## Discussion

We identified five proteins (ST2, IL-6, PTX3, IL-10, renin, and MB) showing a positive relationship with infarct size or negative with ejection fraction and four (TRAIL, TRANCE, IL-16, and CSTB) with elevated values corresponding to higher ejection fraction, smaller infarct, or improved stroke volume. The proteins with most evident associations were IL-6 and ST2.

Our study confirms previous experimental results, links findings to the clinical setting as well as adding to knowledge of the relative importance of these proteins, not possible with single marker studies. Among studied proteins, ST2 and IL–6 were unique in their correlation with early and final infarct size in both univariable and multivariable analyses. IL–6 was also the only protein to show a link with both early and final ejection fraction, emphasizing its role and importance in the post-infarcted myocardium. IL-6 is a pleiotropic interleukin with both pro- and anti-inflammatory effects, involved in T-cell activation and induction of a pro-inflammatory cascade. In STEMI settings, IL-6 has been shown to predict short- and long-term mortality as well as progression to heart failure^18–20^, and our study also confirms previous reports of IL–6 correlation with infarct size and ejection fraction^7^. Besides IL-6, Renin was the only other protein with levels negatively related to long-term ejection fraction after STEMI (Fig. 1). Whereas the inhibition of the renin–angiotensin–aldosterone pathway is an established therapy in heart failure, the role of IL-6 has not been fully elucidated. Recent research has shown elevated levels of IL-6 in 56% of patients with heart failure and an association with poorer clinical outcome in this setting^21^. It has been suggested that IL-6 induces overexpression of angiotensin 2 type 1 receptors in vascular smooth muscle, resulting in oxidative stress and endothelial dysfunction; possibly linking these findings^22^. Direct inhibition of renin in experimental models protects against isoproterenol-induced MI, reperfusion injury and the renin–angiotensin–aldosterone system have also been reported involved in fibroblast scarring and activation of infarct myofibroblasts^9^.

ST2, well-studied in cardiovascular settings, showed a strong correlation with infarct size and was the only protein negatively associated with myocardial salvage index. ST2 is a member of the IL-1 receptor family and exists in a transmembrane isoform and in a soluble isoform. IL-33 is the functional ligand of the ST2 transmembrane isoform, and the IL-33/ST2 signalling pathway results in an immune response through nuclear factor kappa B signalling^23^. In experimental studies, knockout of the IL-33 gene has resulted in increased cardiac remodelling^24^. Administration of recombinant IL-33, on the other hand, has been shown to reduce infarct size and improve ventricular function, an effect mediated through ST2 signalling, as IL-33 does not improve outcome in ST2-knockout mice^25^. The soluble form of ST2, measured in this study, antagonizes the binding of IL-33, functioning as a decoy receptor for IL-33^26^. Elevated levels of soluble ST2 have been observed in patients with poor outcome after MI related to cardiac fibrosis and LV remodelling^27,28^. An interesting finding is that despite the strong link of ST2 levels to infarct size in our study, we observed no correlation with ejection fraction or ∆ ejection fraction, ∆ LVM, or ∆ stroke volume, surrogate measures of LV remodelling^27,28^. The chemo-attractant protein IL-16 and cathepsin enzyme inhibitor CSTB were the only proteins found elevated in patients with improved stroke volume. Elevated levels of IL-16 in patients with STEMI have been previously linked to cardiac fibrosis^29,30^. Considering the pro-inflammatory role of IL-16 as a chemoattractant, this finding is contradictory. However, the complexities of the balance of pro- and anti-inflammation in the acute and subacute phase of STEMI are largely unknown.

PTX, an acute phase reactant, was a factor in early infarct size in both the univariable and multivariable analyses. PTX3, a long pentraxin belonging to the same family of proteins as C-reactive protein, is believed to be an important regulator of inflammation and cardio-protection^31^. In humans, high levels of PTX3 have been observed after MI^32^, and, in experimental studies, PTX3-deficient mice show larger infarcts and greater no-flow area^33^. Proteins with less well established connections to outcome after STEMI were also identified, including TRAIL and TRANCE. TRAIL was negatively correlated to infarct size at 6 months; a finding in line with previous studies showing low levels of TRAIL in patients with poorer outcome post-MI^34^. Finally, we observed levels of IL-10, an anti-inflammatory cytokine with the ability to inhibit release of pro-inflammatory cytokines by macrophages, to be elevated in patients with larger infarcts. Previously, high levels of IL-10 have been found in heart failure after acute myocardial infarction^35,36^.

The findings presented in this study highlight the difficulty of interpreting results of single protein studies in patients with STEMI due to the large amount of proteins that are altered and because of their pleiotropic effects. Whether these proteins are markers of myocardial damage, mediators of processes occurring in the infarcted myocardium or both is yet to be established. Many proteins inversely related to outcome in clinical studies are, nonetheless, associated with smaller infarcts, improved ejection fraction, and reduced LV remodelling in loss-of-function models^33,37,38^. However, our results indicate that, among hundreds of circulating proteins not routinely assayed in clinical setting of MI, IL-6 and ST2 appear to potentially carry valuable prognostic information. Ongoing randomized trials of tocilizumab, a monoclonal antibody targeting IL-6 receptors may add further insight^39^.

This study has a number of limitations. We studied the levels of a large number of proteins relative to several endpoints. This confers a risk of type I error that was controlled for by adjusting for false discovery rate. The OLINK assay is highly sensitive but was unable to detect a number of important proteins. The data are presented as relative units, rendering it difficult to compare results to other studies. Our younger study population with relatively few comorbidities and first-time STEMI, excluding patients with cardiogenic shock at presentation, may not have been entirely representative of the general STEMI population, but provided fewer comorbidity-confounded protein values. Analyses of ejection fraction at 6 months, ∆ left ventricular mass, ∆ ejection fraction, and ∆ stroke volume might have been confounded by discharge medications such as ACE-inhibitors, as they improve ejection fraction as well as reduce remodelling. However, a study without these medications cannot be performed. We have previously shown minor differences in peak levels of some of the assessed proteins in patients treated with hypothermia as compared to a group receiving standard care, possibly due to only modest cooling^40^. Regardless of the limited effect of hypothermia, we chose to adjust for this treatment arm, possibly influencing results for some proteins.

Among 131 circulating inflammatory and cardiovascular proteins in the acute and sub-acute phase of STEMI, IL-6 and ST2 were the most clearly associated with infarct size and ejection fraction as evaluated by CMR in the acute and long-term phase after STEMI. Our study confirms previous experimental findings and provides a link to the clinical setting as well as adding to current knowledge of the importance of these proteins.

## Supplementary information

Supplementary Information.

## References

[1] Ridker PM (2017). Antiinflammatory therapy with canakinumab for atherosclerotic disease. N. Engl. J. Med..

[2] Tardif JC (2019). Efficacy and safety of low-dose colchicine after myocardial infarction. N. Engl. J. Med..

[3] Ridker PM (2019). Low-dose methotrexate for the prevention of atherosclerotic events. N. Engl. J. Med..

[4] Nidorf SM (2020). Colchicine in patients with chronic coronary disease. N. Engl. J. Med..

[5] Ong SB (2018). Inflammation following acute myocardial infarction: Multiple players, dynamic roles, and novel therapeutic opportunities. Pharmacol. Ther..

[6] Palmerini T (2013). Relation between white blood cell count and final infarct size in patients with ST-segment elevation acute myocardial infarction undergoing primary percutaneous coronary intervention (from the INFUSE AMI trial). Am. J. Cardiol..

[7] Orn S (2009). C-reactive protein, infarct size, microvascular obstruction, and left-ventricular remodelling following acute myocardial infarction. Eur. Heart. J..

[8] Mather AN, Fairbairn TA, Artis NJ, Greenwood JP, Plein S (2013). Relationship of cardiac biomarkers and reversible and irreversible myocardial injury following acute myocardial infarction as determined by cardiovascular magnetic resonance. Int. J. Cardiol..

[9] Prabhu SD, Frangogiannis NG (2016). The biological basis for cardiac repair after myocardial infarction: From inflammation to fibrosis. Circ. Res..

[10] Frangogiannis NG (2014). The inflammatory response in myocardial injury, repair, and remodelling. Nat. Rev. Cardiol..

[11] Gislason GH (2006). Risk of death or reinfarction associated with the use of selective cyclooxygenase-2 inhibitors and nonselective nonsteroidal antiinflammatory drugs after acute myocardial infarction. Circulation.

[12] Timmers L (2007). Cyclooxygenase-2 inhibition increases mortality, enhances left ventricular remodeling, and impairs systolic function after myocardial infarction in the pig. Circulation.

[13] Kulasingam A, Hvas AM, Grove EL, Funck KL, Kristensen SD (2018). Detection of biomarkers using a novel proximity extension assay in patients with ST-elevation myocardial infarction. Thromb. Res..

[14] Wilsgaard T (2015). Clinically significant novel biomarkers for prediction of first ever myocardial infarction: The Tromso Study. Circ. Cardiovasc. Genet..

[15] Gidlof O (2019). Proteomic profiling of extracellular vesicles reveals additional diagnostic biomarkers for myocardial infarction compared to plasma alone. Sci. Rep..

[16] Erlinge D (2014). Rapid endovascular catheter core cooling combined with cold saline as an adjunct to percutaneous coronary intervention for the treatment of acute myocardial infarction. The CHILL-MI trial: A randomized controlled study of the use of central venous catheter core cooling combined with cold saline as an adjunct to percutaneous coronary intervention for the treatment of acute myocardial infarction. J. Am. Coll. Cardiol..

[17] Assarsson E (2014). Homogenous 96-plex PEA immunoassay exhibiting high sensitivity, specificity, and excellent scalability. PLoS One.

[18] Wollert KC, Drexler H (2001). The role of interleukin-6 in the failing heart. Heart Fail. Rev..

[19] Borrayo-Sanchez G (2010). Prognostic value of serum levels of interleukin-6 in patients with ST-segment elevation acute myocardial infarction. Cir Cir.

[20] Tan J, Hua Q, Gao J, Fan ZX (2008). Clinical implications of elevated serum interleukin-6, soluble CD40 ligand, metalloproteinase-9, and tissue inhibitor of metalloproteinase-1 in patients with acute ST-segment elevation myocardial infarction. Clin. Cardiol..

[21] Markousis-Mavrogenis G (2019). The clinical significance of interleukin-6 in heart failure: Results from the BIOSTAT-CHF study. Eur. J. Heart Fail..

[22] Wassmann S (2004). Interleukin-6 induces oxidative stress and endothelial dysfunction by overexpression of the angiotensin II type 1 receptor. Circ. Res..

[23] Schmitz J (2005). IL-33, an interleukin-1-like cytokine that signals via the IL-1 receptor-related protein ST2 and induces T helper type 2-associated cytokines. Immunity.

[24] Veeraveedu PT (2017). Ablation of IL-33 gene exacerbate myocardial remodeling in mice with heart failure induced by mechanical stress. Biochem. Pharmacol..

[25] Seki K (2009). Interleukin-33 prevents apoptosis and improves survival after experimental myocardial infarction through ST2 signaling. Circ. Heart Fail..

[26] Pusceddu I, Dieplinger B, Mueller T (2019). ST2 and the ST2/IL-33 signalling pathway-biochemistry and pathophysiology in animal models and humans. Clin. Chim. Acta.

[27] Shimpo M (2004). Serum levels of the interleukin-1 receptor family member ST2 predict mortality and clinical outcome in acute myocardial infarction. Circulation.

[28] Weir RA (2010). Serum soluble ST2: A potential novel mediator in left ventricular and infarct remodeling after acute myocardial infarction. J. Am. Coll. Cardiol..

[29] Schernthaner C (2017). Elevated plasma levels of interleukin-16 in patients with acute myocardial infarction. Medicine (Baltimore).

[30] Tamaki S (2013). Interleukin-16 promotes cardiac fibrosis and myocardial stiffening in heart failure with preserved ejection fraction. PLoS One.

[31] Shiraki A (2016). Pentraxin-3 regulates the inflammatory activity of macrophages. Biochem. Biophys. Rep..

[32] Peri G (2000). PTX3, a prototypical long pentraxin, is an early indicator of acute myocardial infarction in humans. Circulation.

[33] Salio M (2008). Cardioprotective function of the long pentraxin PTX3 in acute myocardial infarction. Circulation.

[34] Osmancik P, Teringova E, Tousek P, Paulu P, Widimsky P (2013). Prognostic value of TNF-related apoptosis inducing ligand (TRAIL) in acute coronary syndrome patients. PLoS One.

[35] Ouyang W, Rutz S, Crellin NK, Valdez PA, Hymowitz SG (2011). Regulation and functions of the IL-10 family of cytokines in inflammation and disease. Annu. Rev. Immunol..

[36] Dominguez Rodriguez A, Abreu Gonzalez P, Garcia Gonzalez MJ, Ferrer Hita J (2005). Association between serum interleukin 10 level and development of heart failure in acute myocardial infarction patients treated by primary angioplasty. Rev. Esp. Cardiol..

[37] Muller J (2014). Interleukin-6-dependent phenotypic modulation of cardiac fibroblasts after acute myocardial infarction. Basic Res. Cardiol..

[38] Hartman MH (2016). Inhibition of interleukin-6 receptor in a murine model of myocardial ischemia-reperfusion. PLoS One.

[39] Anstensrud AK (2019). Rationale for the ASSAIL-MI-trial: A randomised controlled trial designed to assess the effect of tocilizumab on myocardial salvage in patients with acute ST-elevation myocardial infarction (STEMI). Open Heart.

[40] Mohammad MA (2017). Proteomics in hypothermia as adjunctive therapy in patients with ST-segment elevation myocardial infarction: A CHILL-MI substudy. Therap. Hypotherm. Temp. Manag..

