# Supplementary material for: Using proximity extension proteomics assay to identify biomarkers associated with infarct size and ejection fraction after ST-elevation myocardial infarction
Source: Sci Rep. 2020 Oct 29;10:18663. doi: 10.1038/s41598-020-75399-6 (PMC7596042; doi:10.1038/s41598-020-75399-6)
Supplement: Supplementary file 1 — Supplementary Information. [file 41598_2020_75399_MOESM1_ESM.pdf]

Using proximity extension proteomics assay to discover novel biomarkers associated with infarct size and ejection fraction after ST-elevation myocardial infarction

Moman A. Mohammad[1], Sasha Koul[1], Anna Egerstedt[1], J. Gustav Smith[1], Marko Noc[2], Irene Lang[3], Michael Holzer[4], Peter Clemmensen[5], Olof Gidlöf[1], Bernhard Metzler[6], Thomas Engstrøm[7], David Erlinge[1]

1. Department of Cardiology, Clinical Sciences, Lund University, Skane University Hospital, Lund, Sweden
2. Center for Intensive Internal Medicine, Ljubliana, Slovenia
3. Department of Cardiology, Medical University of Vienna, Vienna, Austria
4. Department of Emergency Medicine, Medical University of Vienna, Vienna, Austria
5. Department of General and Interventional Cardiology, University Heart Center, Hamburg-Eppendorf, Hamburg, Germany

Department of Medicine, Division of Cardiology, Nykoebing F Hospital, University of Southern Denmark, Odense, Denmark

6. Department of Cardiology, Innsbruck, Austria
7. The Heart Center, Rigshospitalet, University of Copenhagen, Denmark

Running Title: Proteomics in ST-elevation Myocardial Infarction

Word count: 4982 (3104 excluding references, tables, figure legends).

Keywords: Proteomics, Biomarkers, Infarct size, Ejection fraction, Myocardial Infarction

Corresponding author: David Erlinge, Department of Cardiology, Clinical Sciences, Lund University, Skane University Hospital, Lund; 221 85 Lund, Sweden; telephone: +46 704 872194; fax: +46 46 157857; email: david.erlinge@med.lu.se

Supplementary files

Supplementary Table 1.

CVC panel I

Protein	UniProt No	LOD	LLOQ	ULOQ	Intra-assay	Inter-assay
Adrenomedullin (AM)	P35318	977	977	62500	10%	18%
Agouti-related protein (AGRP)	O00253	244	244	125000	7%	12%
Angiopoietin-1 receptor (TIE2)	Q02763	61	122	250000	8%	12%
Beta-nerve growth factor (Beta-NGF)	P01138	2	2	31250	8%	25%
Caspase-8 (CASP-8)	Q14790	2	2	62500	7%	21%
Cathepsin D (CTSD)	P07339	9766	9766	2500000	8%	16%
Cathepsin L1 (CTSL1)	P07711	244	488	250000	7%	14%
C-C motif chemokine 3 (CCL3)	P10147	0.24	0.48	977	10%	18%
C-C motif chemokine 4 (CCL4)	P13236	0.95	0.95	62500	8%	12%
C-C motif chemokine 20 (CCL20)	P78556	4	4	15630	8%	9%
CD40 ligand (CD40L)	P29965	8	8	7810	8%	16%
Chitinase-3-like protein 1 (CHI3L1)	P36222	488	977	500000	11%	15%
C-X-C motif chemokine 1 (CXCL1)	P09341	15	15	15630	6%	13%
C-X-C motif chemokine 6 (CXCL6)	P80162	61	61	31250	8%	12%
C-X-C motif chemokine 16 (CXCL16)	Q9H2A7	488	488	125000	10%	14%
Cystatin-B (CSTB)	P04080	488	488	250000	8%	13%
Dickkopf-related protein 1 (Dkk-1)	O94907	122	244	250000	8%	15%
Endothelial cell-specific molecule 1 (ESM-1)	Q9NQ30	244	488	250000	9%	16%
Eosinophil cationic protein (ECP)	P12724	488	488	15630	5%	22%
Epidermal growth factor EGF)	P01133	0.95	2	3910	5%	9%
E-selectin (SELE)	P16581	244	488	500000	9%	13%
Fatty acid-binding protein, adipocyte (FABP4)	P15090	977	1950	250000	12%	14%
Fibroblast growth factor 23 FGF-23)	Q9GZV9	122	488	62500	9%	21%
Follistatin (FS)	P19883	977	977	1000000	10%	13%
Fractalkine (CX3CL1)	P78423	15	15	62500	9%	14%
Galanin peptides (GAL)	P22466	488	488	1000000	9%	17%
Galectin-3 (Gal-3)	P17931	3906	3906	500000	9%	12%
Growth hormone (GH)	P01241	0.95	2	15630	5%	14%
Growth/differentiation factor 15 (GDF-15)	Q99988	8	15	62500	9%	11%
Heat shock 27 kDa protein (HSP 27)	P04792	3910	7810	500000	9%	13%
Heparin-binding EGF-like growth factor (HB-EGF)	Q99075	2	2	7810	5%	14%
Hepatocyte growth factor (HGF)	P14210	31	61	125000	7%	12%
Interleukin-1 receptor antagonist protein (IL-1ra)	P18510	4	4	1950	6%	14%
Interleukin-4 (IL-4)	P05112	0.95	0.95	15630	5%	11%
Interleukin-6 (IL-6)	P05231	0.06	0.06	3910	8%	10%
Interleukin-6 receptor subunit alpha (IL-6RA)	P08887	488	488	500000	8%	14%
Interleukin-8 (IL-8)	P10145	0.12	0.12	3910	8%	12%
Interleukin-16 (IL-16)	Q14005	15	15	15630	5%	11%

Interleukin-18 (IL-18)	Q14116	0.24	0.24	31250	8%	12%
Interleukin-27 (IL-27)	Q8NEV9, Q14213	122	122	62500	8%	19%
Kallikrein-6 (KLK6)	Q92876	8	8	62500	11%	22%
Kallikrein-11 (hk11)	Q9UBX7	8	8	15630	9%	19%
Lectin-like oxidized LDL receptor 1 (LOX-1)	P78380	8	8	15630	5%	12%
Leptin (LEP)	P41159	488	488	62500	8%	12%
Macrophage colony-stimulating factor 1 (CSF-1)	P09603	0.12	0.12	7810	7%	12%
Matrix metalloproteinase-1 (MMP-1)	P03956	4	4	31250	5%	31%
Matrix metalloproteinase-3 (MMP-3)	P08254	3910	3910	250000	7%	20%
Matrix metalloproteinase-7 (MMP-7)	P09237	31	61	62500	11%	16%
Matrix metalloproteinase-10 (MMP-10)	P09238	4	4	31250	7%	15%
Matrix metalloproteinase-12 (MMP-12)	P39900	8	8	125000	9%	39%
Melusin (ITGB1BP2)	Q9UKP3	122	122	125000	13%	21%
Membrane-bound aminopeptidase P (mAmP)	O43895	9766	9766	1250000	13%	22%
Monocyte chemotactic protein 1 (MCP-1)	P13500	2	4	1950	7%	18%
Myeloperoxidase (MPO)	P05164	488	488	250000	5%	18%
Myoglobin (MB)	P02144	4883	4883	1250000	9%	17%
Natriuretic peptides B (BNP)	P16860	NR	NR	NR	NR	NR
NF-kappa-B essential modulator (NEMO)	Q9Y6K9	NR	NR	NR	8%	20%
N-terminal pro-B-type natriuretic peptide (NT-proBNP)	NA	3910	3910	250000	NR	NR
Osteoprotegerin (OPG)	O00300	0.95	0.95	62500	7%	11%
Ovarian cancer-related tumor marker CA 125 (CA-125)	Q8WXI7	NR	NR	NR	10%	20%
Pappalysin-1 (PAPPA)	Q13219	4883	9766	10000000	21%	5%
Pentraxin-related protein PTX3 (PTX-3)	P26022	244	244	15630	8%	20%
Placenta growth factor (PlGF)	P49763	0.48	2	31250	8%	13%
Platelet endothelial cell adhesion molecule (PECAM-1)	P16284	244	244	62500	8%	16%
Platelet-derived growth factor subunit B (PDGF subunit B)	P01127	244	244	250000	9%	14%
Prolactin (PRL)	P01236	3906	7812	1000000	9%	14%
Protein S100-A12 (EN-RAGE)	P80511	244	244	1000000	8%	22%
Proteinase-activated receptor 1 (PAR-1)	P25116	NR	NR	NR	6%	14%
Proto-oncogene tyrosine-protein kinase Src (SRC)	P12931	76	76	78125	4%	12%
P-selectin glycoprotein ligand 1 (PSGL-1)	Q14242	61	61	31250	8%	20%
Receptor for advanced glycosylation end products (RAGE)	Q15109	15	31	31250	8%	13%
Renin (REN)	P00797	15	15	31250	7%	13%
Resistin (RETN)	Q9HD89	122	244	250000	9%	18%
SIR2-like protein (SIRT2)	Q8IXJ6	122	122	125000	11%	20%
Spondin-1 (SPON1)	Q9HCB6	3906	3906	1000000	10%	13%
ST2 protein (ST2)	Q01638	122	122	125000	9%	14%
Stem cell factor (SCF)	P21583	0.12	0.12	7810	6%	13%
Thrombomodulin (TM)	P07204	8	15	15625	8%	14%
TIM-1 (TIM)	Q96D42	8	8	31250	10%	14%
Tissue factor (TF)	P13726	0.06	0.12	7810	7%	16%
Tissue-type plasminogen activator (t-PA)	P00750	244	244	500000	9%	14%

TNF-related activation-induced cytokine (TRANCE)	O14788	15	15	125000	9%	16%
TNF-related apoptosis-inducing ligand (TRAIL)	P50591	4	4	31250	7%	15%
TNF-related apoptosis-inducing ligand receptor 2 (TRAIL-R2)	O14763	8	15	62500	7%	15%
Tumor necrosis factor receptor 1 (TNF-R1)	P19438	2	4	31250	7%	11%
Tumor necrosis factor receptor 2 (TNF-R2)	P20333	488	488	250000	9%	14%
Tumor necrosis factor receptor superfamily member 5 (CD40)	P25942	0.24	0.48	15630	7%	12%
Tumor necrosis factor receptor superfamily member 6 (FAS)	P25445	31	31	250000	8%	12%
Tumor necrosis factor ligand superfamily member 14 (TNFSF14)	O43557	2	2	31250	8%	25%
Urokinase plasminogen activator surface receptor (uPA)	Q03405	0.24	0.48	7810	5%	12%
Vascular endothelial growth factor A (VEGF-A)	P15692	0.12	0.24	15630	8%	13%
Vascular endothelial growth factor D (VEGF-D)	O43915	61	61	62500	7%	13%

INF I panel

Protein	UniProt No	LOD	LLOQ	ULOQ	Intra	Inter
Adenosine Deaminase (ADA)	P00813	0.48	0.48	31250	5%	29%
Artemin (ARTN)	Q5T4W7	0.24	0.48	31250	7%	18%
Axin-1 (AXIN1)	O15169	61	61	62500	6%	19%
Beta-nerve growth factor (Beta-NGF)	P01138	0.48	0.48	15625	6%	14%
Brain-derived neurotrophic factor (BDNF)	P23560				6%	10%
Caspase 8 (CASP-8)	Q14790	0.48	0.48	31250	7%	22%
C-C motif chemokine 4 (CCL4)	P13236	1.9	1.9	31250	6%	17%
C-C motif chemokine 19 (CCL19)	Q99731	15	15	31250	8%	15%
C-C motif chemokine 20 (CCL20)	P78556	7.6	7.6	15625	7%	13%
C-C motif chemokine 23 (CCL23)	P55773	31	31	31250	6%	13%
C-C motif chemokine 25 (CCL25)	O15444	3.8	3.8	62500	6%	18%
C-C motif chemokine 28 (CCL28)	Q9NRJ3	61	122	1000000	7%	14%
CD40L receptor (CD40)	P25942	0.01	0.01	3906	5%	21%
CUB domain-containing protein 1 (CDCP1)	Q9H5V8	0.12	0.12	7812	6%	24%
C-X-C motif chemokine 1 (CXCL1)	P09341	3.8	7.6	15625	6%	15%
C-X-C motif chemokine 5 (CXCL5)	P42830	0.95	0.95	7812	7%	13%
C-X-C motif chemokine 6 (CXCL6)	P80162	7.6	31	15625	8%	14%
C-X-C motif chemokine 9 (CXCL9)	Q07325	0.95	0.95	3906	6%	12%
C-X-C motif chemokine 10 (CXCL10)	P02778	7.6	7.6	15625	7%	11%
C-X-C motif chemokine 11 (CXCL11)	O14625	7.6	31	15625	7%	14%
Cystatin D (CST5)	P28325	1.9	1.9	15625	5%	21%
Delta and Notch-like epidermal growth factor- related recep (DNER)	Q8NFT8	0.95	1.9	31250	5%	26%
Eotaxin-1 (CCL11)	P51671	3.8	3.8	31250	5%	14%
Eukaryotic translation initiation factor 4E-binding	Q13541				6%	23%
Fibroblast growth factor 5 (FGF-5)	Q8NF90	1.9	1.9	31250	7%	14%
Fibroblast growth factor 19 (FGF-19)	O95750	7.6	7.6	15625	6%	19%
Fibroblast growth factor 21 (FGF-21)	Q9NSA1	31	31	62500	8%	21%
Fibroblast growth factor 23 (FGF-23)	Q9GZV9	122	122	62500	9%	26%
Fms-related tyrosine kinase 3 ligand (Flt3L)	P49771	0.01	0.01	977	6%	15%

Fractalkine (CX3CL1)	P78423	15.3	15.3	15625	7%	24%
Glial cell line-derived neurotrophic factor (hGDNF)	P39905	0.01	0.01	1953	7%	12%
Hepatocyte growth factor (HGF)	P14210	7.6	7.6	125000	6%	16%
Interferon gamma (IFN-gamma)	P01579	15.3	15.3	62500	7%	17%
Interleukin-1 alpha (IL-1 alpha)	P01583	0.48	0.95	31250	7%	18%
Interleukin-2 (IL-2)	P60568	30.5	30.5	1000000	9%	16%
Interleukin-2 receptor subunit beta (IL-2RB)	P14784	15	31	250000	7%	19%
Interleukin-4 (IL-4)	P05112	0.24	0.24	7812	7%	16%
Interleukin-5 (IL-5)	P05113	3.8	3.8	15625	7%	17%
Interleukin-6 (IL-6)	P05231	0.12	0.12	3906	6%	8%
Interleukin-7 (IL-7)	P13232	0.24	0.24	7812	6%	18%
Interleukin-8 (IL-8)	P10145	0.03	0.03	3906	6%	15%
Interleukin-10 (IL-10)	P22301	0.48	0.48	62500	7%	12%
Interleukin-10 receptor subunit alpha (IL-10RA)	Q13651	3.8	7.6	250000	6%	19%
Interleukin-10 receptor subunit beta (IL-10RB)	Q08334	0.12	0.12	1953	5%	31%
Interleukin-12 subunit beta (IL-12B)	P29460	0.12	0.12	3906	6%	16%
Interleukin-13 (IL-13)	P35225	7.6	7.6	62500	14%	26%
Interleukin-15 receptor subunit alpha (IL-15RA)	Q13261	0.95	0.95	7812	6%	20%
Interleukin-17A (IL-17A)	Q16552	3.8	7.6	62500	8%	17%
Interleukin-17C (IL-17C)	Q9P0M4	31	31	125000	8%	18%
Interleukin-18 (IL-18)	Q14116	0.06	0.06	15625	6%	19%
Interleukin-18 receptor 1 (IL-18R1)	Q13478	0.06	0.06	7812	5%	26%
Interleukin-20 (IL-20)	Q9NYY1	7.6	15	62500	7%	22%
Interleukin-20 receptor subunit alpha (IL-20RA)	Q9UHF4	1.9	1.9	125000	6%	22%
Interleukin-22 receptor subunit alpha-1 (IL-22 RA1)	Q8N6P7	0.24	0.24	3906	7%	23%
Interleukin-24 (IL-24)	Q13007	1.9	3.8	31250	6%	29%
Interleukin-33 (IL-33)	O95760	3.8	3.8	31250	9%	26%
Latency-associated peptide transforming growthfactor beta 1 (LAP TGF-beta-1)	P01137	61	61	500000	7%	24%
Leukemia inhibitory factor (LIF)	P15018	3.8	7.6	15625	7%	18%
Leukemia inhibitory factor receptor (LIF-R)	P42702	30.5	15.3	62500	7%	26%
Macrophage colony-stimulating factor 1 (CSF-1)	P09603	0.004	0.01	1953	5%	25%
Macrophage inflammatory protein 1-alpha (MIP-1)	P10147	0.06	0.06	488	6%	14%
Matrix metalloproteinase-1 (MMP-1)	P03956	1.9	3.8	15625	5%	19%
Matrix metalloproteinase-10 (MMP-10)	P09238	0.95	0.95	15625	5%	28%
Monocyte chemotactic protein 1 (MCP-1)	P13500	0.03	0.03	1953	6%	13%
Monocyte chemotactic protein 2 (MCP-2)	P80075	0.06	0.06	3906	6%	8%
Monocyte chemotactic protein 3 (MCP-3)	P80098	0.48	0.48	1953	7%	17%
Monocyte chemotactic protein 4 (MCP-4)	Q99616	7.6	7.6	3906	6%	11%
Natural killer cell receptor 2B4 (CD244)	Q9BZW8	0.06	0.06	7812	5%	24%
Neurotrophin-3 (NT-3)	P20783	0.12	0.12	3906	6%	13%
Neurturin (NRTN)	Q99748	3.8	7.6	15625	9%	15%
Oncostatin-M (OSM)	P13725	0.03	0.03	977	5%	12%
Osteoprotegerin (OPG)	O00300	0.24	0.48	31250	6%	12%

Programmed cell death 1 ligand 1 (PD-L1)	Q9NZQ7	3.8	3.8	500000	9%	25%
Protein S100-A12 (EN-RAGE)	P80511	122	122	500000	8%	17%
(SSiLgnAaMliFn1g) lymphocytic activation molecule	Q13291	31	31	1000000	9%	21%
SIR2-like protein 2 (SIRT2)	Q8IXJ6	7.6	15.3	62500	8%	22%
STAM-binding protein (STAMPB)	O95630	7.6	7.6	31250	5%	27%
Stem cell factor (SCF)	P21583	1.9	3.8	15625	5%	20%
Sulfotransferase 1A1 (ST1A1)	P50225	244	244	125000	6%	25%
T-cell surface glycoprotein CD5 (CD5)	P06127	0.06	0.12	3906	5%	22%
T cell surface glycoprotein CD6 isoform (CD6)	Q8WWJ7	0.24	0.24	7812	6%	23%
Thymic stromal lymphopoietin (TSLP)	Q969D9	3.8	3.8	15625	6%	20%
TNF-beta (TNFB)	P01374	0.24	0.48	15625	6%	22%
TNF-related activation-induced cytokine (TRANCE)	O14788	3.8	3.8	31250	7%	24%
TNF-related apoptosis-inducing ligand (TRAIL)	P50591	0.95	0.95	31250	5%	17%
Transforming growth factor alpha (TGF-alpha)	P01135	0.48	0.48	3906	6%	27%
Tumor necrosis factor (Ligand) superfamily,member 12 (TWEAK)	Q4ACW9	1.9	1.9	125000	6%	11%
Tumor necrosis factor (TNF)	P01375	0.48	0.48	3906	9%	11%
T1u4 m(TorN FnSeFcr1o4s)is factor ligand superfamily member	O43557	0.95	1.9	15625	6%	15%
Tumor necrosis factor receptor superfamilymember 9 (TNFRSF9)	Q07011	0.03	0.03	3906	5%	21%
Urokinase-type plasminogen activator (uPA)	P00749	0.12	0.12	7812	5%	11%
Vascular endothelial growth factor A (VEGF-A)	P15692	0.06	0.06	7812	6%	8%
